# Association of urinary concentrations of early pregnancy phthalate metabolites and bisphenol A with length of gestation

**DOI:** 10.1186/s12940-019-0522-2

**Published:** 2019-08-30

**Authors:** Helen B. Chin, Anne Marie Jukic, Allen J. Wilcox, Clarice R. Weinberg, Kelly K. Ferguson, Antonia M. Calafat, D. Robert McConnaughey, Donna D. Baird

**Affiliations:** 1 Epidemiology Branch, National Institute of Environmental Health Sciences, 111 T W Alexander Drive, Mailstop A3-05, Research Triangle Park, NC 27709 USA; 2 Biostatistics Branch, National Institute of Environmental Health Sciences, 111 T W Alexander Drive, Research Triangle Park, NC 27709 USA; 3 Division of Laboratory Sciences, National Center for Environmental Health, Centers for Disease Control and Prevention, Mail Stop F-17, 4770 Buford Highway, NE, Atlanta, GA 30341-3724 USA; 4 Westat, 1009 Slater Rd # 110, Durham, NC 27703 USA

**Keywords:** Pregnancy, Pregnancy length, Delivery

## Abstract

**Background:**

Environmental exposure to phthalates and bisphenol A (BPA) may have endocrine disrupting effects that alter length of gestation. We assessed the association between the urinary concentrations of 11 phthalate metabolites and BPA with length of gestation in a cohort of women followed from before conception with daily 1st-morning urinary hormone measures that identified day of implantation.

**Methods:**

Pre-implantation and post-implantation urinary phthalate metabolites and BPA concentrations were measured in pooled urine samples designed to limit single-measure variability due to the likely episodic nature of these exposures and the short half-life of these compounds. We estimated associations between these exposure biomarkers early in pregnancy with length of gestation from implantation to spontaneous birth. Cox proportional hazards models were used to estimate the hazard of birth among 125 naturally-conceived, singleton live births with censoring for medical interventions that artificially shortened pregnancy.

**Results:**

Higher concentrations of mono (2-ethyl-5-hydroxyhexyl) phthalate (a metabolite of di (2-ethylhexyl) phthalate (DEHP)) during the pre-implantation window were associated with reduced probability of birth, i.e., longer gestations (hazard ratio (HR): 0.55, 95% CI: 0.35, 0.86; *p* = 0.01). The HR for the molar sum of the four DEHP metabolites measured showed a similar association (HR: 0.67, 95% CI: 0.43, 1.05). Higher concentrations of mono (3-carboxypropyl) phthalate (MCPP), a non-specific metabolite of several high molecular-weight phthalates, measured post-implantation were associated with increased risk of earlier birth, i.e. shorter length of gestation, HR: 1.59, CI: 1.02, 2.49.

**Conclusions:**

Early gestational exposure to DEHP and possibly other high-molecular weight phthalates, (as reflected by urinary MCPP concentrations) may influence the length of pregnancy. Such effects could have consequences for neonatal and maternal health.

**Electronic supplementary material:**

The online version of this article (10.1186/s12940-019-0522-2) contains supplementary material, which is available to authorized users.

## Background

There is widespread exposure to phthalates and bisphenol A (BPA) in the U.S. population [1–3]. Phthalates are used to increase flexibility of plastics, are in food packaging and medical devices, and are also components of many personal care products [4, 5]. Human exposure to phthalates occurs through ingestion, inhalation, and dermal contact [3]. BPA is used widely as a component of hard plastics and epoxy resins and human exposure occurs primarily through ingestion of contaminated food and water [3]. Phthalates have been shown to disrupt both estrogen and androgen mediated processes [6] and BPA can act as an estrogen [7] although with a lower binding affinity than estradiol [8]. Phthalates have also been shown to be associated with increased oxidative stress biomarkers during pregnancy [9]. Because of the endocrine disrupting properties of phthalates and BPA and their potential to increase oxidative stress, exposure could plausibly interfere with the hormonal processes of pregnancy and affect the developing fetus.

There is natural variability in the length of human gestation [10], but preterm birth, or birth before 37 completed weeks of gestation and post-term birth (after 42 weeks gestation) are a public health concern given the increased risk of neonatal morbidity and mortality in both groups [11, 12]. Studies also show adverse effects of births before 38 or 39 completed weeks [13–15]. Studies that have examined the association between phthalate exposure and length of gestation report inconsistent findings [16–19]. Fewer studies have examined the association between BPA exposure and length of gestation. These studies focus on preterm birth as the outcome and also report inconsistent findings [20, 21]. In the North Carolina Early Pregnancy Study (EPS), a cohort of naturally conceiving women, early pregnancy events were predictive of length of gestation. These early events of pregnancy were hormonally defined and included the number of days between ovulation and embryo implantation and timing and pattern of post-implantation rise in progesterone (rescue of the corpus luteum) [10]. These findings suggest that characteristics of the hormonal changes necessary to maintain early pregnancy can be associated with gestational length. To further investigate early pregnancy exposures, we conducted an analysis to assess associations of urinary concentrations of phthalate biomarkers and BPA measured in early pregnancy with length of gestation in this cohort of women with no known fertility problems.

## Methods

### Study description

The EPS was a prospective cohort study conducted in 1982–1986 to estimate the incidence of early pregnancy loss [22, 23]. Briefly, women with no known fertility problems enrolled at the time they discontinued birth control to become pregnant (*n* = 221) and kept daily diaries in which they recorded sexual intercourse and menstrual bleeding. Women were followed for up to 6 months for the occurrence of a clinically-recognized pregnancy. Daily first-morning urine specimens were collected in screw top polypropylene jars without preservative throughout the study. For women with a clinically-recognized pregnancy (*n* = 151), urine collection continued until 8 weeks after their last menstrual period (LMP). Urine specimens were used to measure reproductive hormones to characterize the events of early pregnancy including day of implantation [22]. Women were contacted after their estimated delivery date to gather information on the outcome of their pregnancies including delivery date. Participants were re-contacted in 2010–2011 and 173 of 210 living participants completed a follow-up questionnaire which included questions about the method of delivery of their EPS baby for those who had a live birth. This information was used to identify women who underwent a medical intervention that had artificially shortened their pregnancy (induced labor or Caesarean delivery without labor) [10]. The study protocol was approved by the Institutional Review Board of the National Institute of Environmental Health Sciences.

### Exposure assessment

Urine samples were stored for up to 2 weeks in participants’ freezers before collection by study staff and storage at a central location at − 20 °C. After pilot testing to assess the stability of urinary phthalate metabolites and BPA during the storage period [24, 25], concentrations were measured in the stored urine samples [26]. These compounds have a relatively short half-life [27, 28] and participants likely had episodic exposure. To provide a more stable and representative estimate of exposure [26] aliquots were pooled from each of three urine specimens from each menstrual cycle and early pregnancy, for those who achieved pregnancy. In most cases Monday samples were used for pooling because the study protocol requested that women collect more urine on Mondays. Two distinct early pregnancy-related pools were made, one for the pre-implantation weeks of the conception cycle and one for the post-implantation pregnancy window. For the pre-implantation window pool, the first 3 Monday samples were selected from the interval starting the day after the end of the LMP (defined as the first two consecutive days of no bleeding after the onset of menses) and ending on the day before implantation was detected (Additional file 1: Figure S1). If there were not 3 Mondays during this interval or a Monday sample was missing, a sample from a neighboring day was used for pooling. The post-implantation samples were selected from the first 3 Monday collections starting on the day after implantation (i.e., samples ranging from 1 to 34 days after initial rise in hCG) (Additional file 1: Figure S2). Again, if 3 Monday samples were not available, substitutions from alternate days of the week were included so that each woman contributed 3 urine specimens to her pooled sample.

Urine specimens were analyzed for phthalate metabolites and BPA at the Centers for Disease Control and Prevention (CDC) using online solid-phase-extraction, high performance liquid chromatography-isotope dilution tandem mass spectrometry [29, 30]. The laboratory quantified 12 chemical biomarkers in all pooled urine samples, namely BPA and 11 phthalate metabolites: mono-n-butyl phthalate (MBP), monoethyl phthalate (MEP), monobenzyl phthalate (MBzP), mono(2-ethylhexyl) phthalate (MEHP), mono(2-ethyl-5-oxohexyl) phthalate (MEOHP), mono(2-ethyl-5-hydroxyhexyl) phthalate (MEHHP), mono(2-ethyl-5-carboxypentyl) phthalate (MECPP), monocarboxynonyl phthalate (MCNP), monocarboxyoctyl phthalate (MCOP), mono(3-carboxypropyl) phthalate (MCPP), and mono-isobutyl phthalate (MiBP) commonly evaluated in epidemiologic studies. None of the measurements from the pooled specimens were below any of the limits of detection. All BPA and phthalate metabolite concentrations were standardized by the creatinine concentration, also measured in the pooled specimen. The involvement of the CDC laboratory did not constitute engagement in human subjects research.

### Outcome measure

Daily urine samples were analyzed for human chorionic gonadotropin (hCG) at the time of the original study (1982–1986) using a highly sensitive immunoradiometric assay with a polyclonal antibody [23]. A sustained rise in hCG was used to identify pregnancy and the day of implantation of the conceptus. An hCG level ≥ 0.025 ng/ml for 3 consecutive days defined the presence of a pregnancy [22]. Among identified pregnancies, the first day of sustained rise ≥0.015 ng/ml was assigned as the day of implantation [31]. Urinary hCG levels were not standardized by the creatinine concentration because the magnitude of rise in hCG was considerably greater than the variations in creatinine, making adjustment unnecessary [32].

Length of gestation was quantified as the number of days from implantation to spontaneous birth, with censoring for early delivery due to medical intervention (i.e., induced labor or Caesarean delivery without labor) [10]. We used the length of gestation from implantation to birth because the alternative LMP-based gestational length would include additional variability due to differences in follicular phase length [10] and variability in the time from ovulation to implantation. The association between the phthalate metabolites and BPA and time from ovulation to implantation has already been examined in this cohort [33].

### Statistical analysis

Analyses were performed using SAS version 9.4 (SAS Inc., Cary, NC). The analysis of gestational length from implantation to birth was limited to singleton live births. We imputed the values for women missing date of implantation (*n* = 6), based on the hCG level on the first non-missing day [10]. We excluded one woman missing phthalate metabolite and BPA measurements and four women exposed to diethylstilbestrol (DES) in utero because DES had been examined previously and found to be associated with irregular hormonal patterns in early pregnancy and increased risk of early birth [10, 34].

BPA and each of the phthalate metabolites measured were assessed in separate models. For the di(2-ethylhexyl) phthalate (DEHP) metabolites (MEHP, MEHHP, MEOHP, MECPP), we also examined associations between their sum-based molar concentration (∑DEHP metabolites in nmol/mL) and length of gestation. Pre-implantation and post-implantation early pregnancy measures of the chemical biomarkers of interest were assessed separately to examine two windows of exposure with potentially distinct effects. The pre-implantation window measures represent exposure around the time of fertilization and the post-implantation measures represent exposure during the first few weeks after implantation. Potential covariates for analysis were identified from prior analysis of participant characteristics and gestational length [10]. We considered maternal characteristics associated with the exposure and outcome as potential confounders and adjusted for maternal age in the analysis. Other maternal characteristics such as body mass index, smoking status, race, and parity were not associated with length of gestation in this cohort.

We used Cox proportional hazards models to estimate the associations between the measured exposure biomarkers and gestational length (time from implantation to spontaneous birth). Gestational time was the primary time scale and exposures were examined as dichotomous (above and below the median) and as tertiles. The tertile analysis did not show evidence of a non-monotonic association, so we present the results from the dichotomized exposure analysis only. In this analysis a hazard ratio greater than 1.0 indicates an increased risk of birth (i.e. shorter gestation) among women with exposure biomarker concentrations above the median. This model accounts for the occurrence of medical interventions before spontaneous labor occurs by censoring these gestations at birth. However, 27 women (22%) were missing data on whether they had such medical interventions. We used multiple imputation methods [35] to impute whether medical interventions that shortened pregnancy occurred for these 27 women. We generated 400 data sets, with a “yes” or “no” value for medical intervention (the censoring variable) [10]. The multiple imputation allowed us to have data on medical interventions that artificially shortened pregnancy (labor induction or Caesarean section without labor) for all of the women in our study, so that we could assess length of natural pregnancy and censor pregnancies that did not end in spontaneous birth. Using this approach, the hazard ratio for each biomarker was obtained as an average of the hazard ratios across imputations. To calculate 95% confidence intervals, the variance of the logarithm of the hazard ratio was estimated using the total number of imputations and the variance between and within the imputations [36]. *P*-values were calculated using a chi-square distribution with 1 degree of freedom where the test statistic was calculated as the mean coefficient estimate across imputations squared divided by the total variance [36]. For exposure biomarkers with hazard ratios indicating longer or shorter gestations, we used a lifetable analysis combining the imputed datasets to compare differences in median days gestation between exposure groups. The difference in the median days of gestation was used as an estimate of the difference in gestational length (number of days longer or shorter length) for women who had above median concentrations of the exposure biomarker compared to women with below median concentrations.

## Results

Our sample included singleton live births to mothers who had not been exposed to DES in utero and had measured urinary BPA and phthalate metabolites, resulting in an analytic sample size of 125 women (Additional file 1: Figure S3). The median age of the women in the study was 28 years. Most women were white (95%), college educated (71%), never smokers (83%), and 66% had a prior pregnancy at enrollment. The median concentrations of phthalate metabolites and BPA for the pre-implantation and post-implantation early pregnancy windows are presented in Table 1. Correlations between the exposure biomarker concentrations between the two windows were relatively low, with Spearman correlation coefficients ranging from 0.22 for MCNP to 0.60 for MCPP *(*Additional file 1: Table S1)*.* Gestational length from implantation to birth ranged from 199 to 275 days. Assuming a 14-day interval from LMP to ovulation and a 9-day interval from ovulation to implantation, this corresponds to a 31.7–42.6 week LMP-based length of pregnancy. Six births were preterm (< 37 weeks from LMP, i.e. ≤236 days from implantation) and 4 births were post-term (> 42 weeks from LMP, i.e. > 271 days from implantation).

**Table 1:** Concentrations of measured biomarkers during the pre-implantation (*n* = 125) and post-implantation (*n * = 121) early pregnancy windows

Biomarker^a^	Pre-implantation (*n* = 125)		Post-implantation (*n* = 121)^b^	
	Median	(IQR)	Median	(IQR)
MBP	58.7	(42.2, 85.6)	54.5	(37.2, 82.9)
MEP	105.4	(56.6, 234.1)	92.5	(53.6, 146.3)
MBzP	34.1	(20.3, 48.8)	25.3	(17.5, 39.4)
MEHP	5.3	(3.3, 7.8)	5.4	(3.3, 8.3)
MEHHP	36.0	(27.9, 55.2)	32.8	(24.2, 48.3)
MEOHP	23.2	(17.8, 31.6)	22.4	(16.2, 32.4)
MECPP	50.2	(39.4, 69.6)	48.1	(37.8, 68.4)
MCNP	2.8	(1.9, 4.5)	3.2	(2.2, 5.7)
MCOP	2.6	(1.9, 3.6)	2.0	(1.4, 2.8)
MCPP	10.5	(7.9, 14.7)	9.9	(6.9, 14.0)
MiBP	2.3	(1.7, 3.6)	2.0	(1.3, 2.8)
∑DEHP^c^	0.4	(0.3, 0.5)	0.4	(0.3, 0.5)
BPA	2.0	(1.4, 3.0)	1.8	(1.1, 2.6)

^a^Creatinine adjusted (ng/mg creatinine).

^b^Four women did not have post-implantation early pregnancy samples.

^c^∑DEHP represents the molar sum of 4 metabolites of DEHP: MEHP, MEHHP, MEOHP, and MECPP creatinine adjusted (nmol/mg creatinine).

**Fig. 1:**
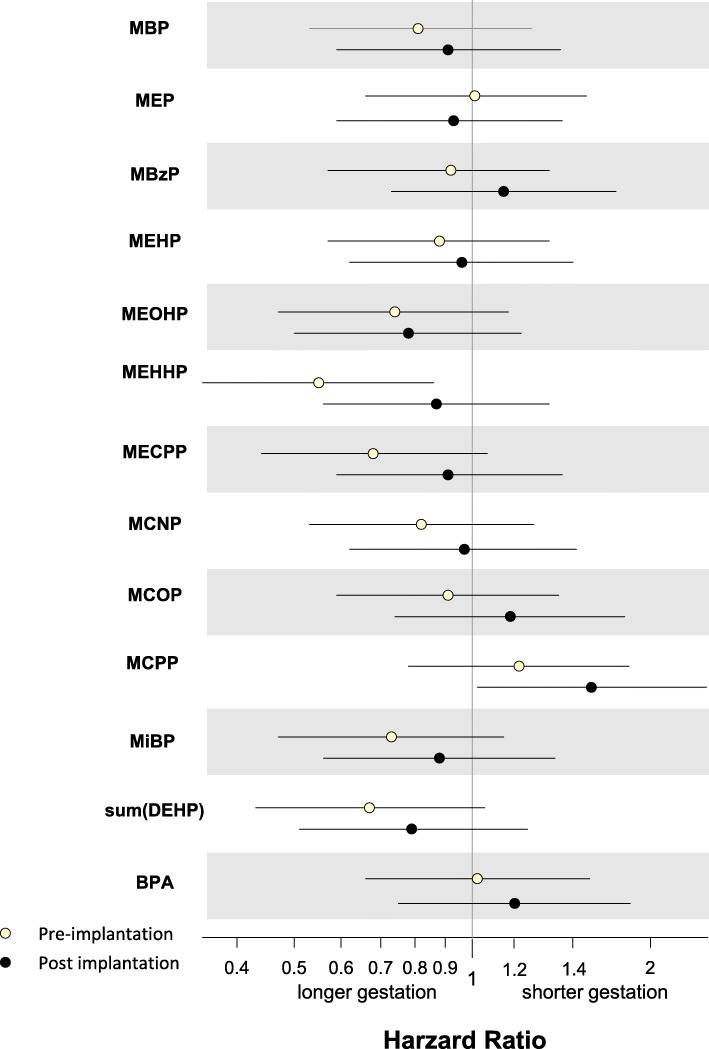
Associations between pre-implantation (*n* = 125) and post-implantation (*n* = 121) early pregnancy biomarker concentrations and implantation-based length of gestation. Pre-implantation (white dots) hazard ratios represent the risk of birth for women with pre-implantation measured biomarker concentrations above the median compared with those below the median (reference) and post-implantation (black dots) hazard ratios represent the risk of birth for women with post-implantation measured biomarker concentrations above the median compared with those below the median (reference). Hazard ratios < 1.0, indicate a reduced risk of delivery (i.e., longer gestation) and hazard ratios > 1.0 indicate increased risk of delivery (i.e, shorter gestation)

Hazard ratios (HR) for the occurrence of birth by exposure biomarker concentrations are presented in Fig. 1 *and* Additional file 1: Table S2*.* Maternal age could theoretically be a confounder in our analysis because it was previously found to be associated with length of gestation in this cohort [10], but because it was unrelated to phthalate and BPA biomarker concentrations, we present only unadjusted estimates. The main findings from our assessment of the association of 11 phthalate metabolites and BPA with implantation-based length of gestation were for the DEHP metabolites and MCPP. All the DEHP metabolites had HRs < 1.0, indicating a reduced risk of delivery (i.e., longer gestation), but estimates were only statistically significant for MEHHP concentrations prior to implantation (HR: 0.55, 95% CI: 0.35, 0.86; *p* = 0.01). This corresponded to an approximate 3 day longer pregnancy among women with higher than median concentrations of MEHHP during the pre-implantation window compared to women with lower concentrations. The HR for the composite measure, ∑DEHP, was 0.67, 95% CI: 0.43, 1.05 (approximate 2 day longer gestation for women with higher than median concentrations) and approached statistical significance (*p* = 0.08). Higher than median concentrations of MCPP during the post-implantation window were associated with increased risk of earlier birth (shorter gestation by approximately 4 days), HR: 1.59, CI: 1.02, 2.49 (*p* = 0.04). A non-significant and attenuated estimate in the same direction was also seen for the pre-implantation MCPP concentrations and length of gestation. Results from the remainder of the chemical biomarkers examined had wide confidence intervals overlapping 1.0, suggesting null associations.

## Discussion

This cohort of naturally conceiving women provided the opportunity to assess how exposure during very early pregnancy (before the pregnancy is recognized) may influence the sequalae of events that occur throughout pregnancy until birth. In the current analysis, we found that the DEHP metabolites all had hazard ratios less than 1.0 indicating longer gestations for women with higher than median concentrations of the 4 measured metabolites (MEHP, MEHHP, MEOHP, MECPP). The largest estimate was for pre-implantation MEHHP and this was also the only statistically significant DEHP-metabolite association. We also observed an elevated HR for post-implantation MCCP, a non-specific metabolite of several high molecular weight phthalates, suggesting increased risk of earlier birth. These results suggest that there may be different effects of phthalate exposure during the pre-implantation window compared with the post-implantation window on length of pregnancy, however overlapping confidence intervals limit our ability to draw conclusions about differences in the associations for each of the time windows. The parent compounds of MEHHP and MCPP are high molecular weight phthalates that are commonly used in food packaging, soft plastics, medications, and cosmetics [37], which represent potential routes of exposure. None of the other phthalate biomarkers evaluated or BPA was associated with length of gestation.

Phthalates and BPA have the potential to interfere with the hormonal changes that occur in early pregnancy [38, 39] and induce oxidative stress and inflammation [40], two pathways through which these compounds could affect overall pregnancy health and length of gestation. Findings from our prior research in this cohort provide context for our current findings. Pre-implantation DEHP metabolites were associated with a decreased frequency of late corpus luteum rescue [33] and reduced risk of early pregnancy loss (loss before 6 weeks gestation) [26], suggestive of better pregnancy health. The association of the pre-implantation DEHP metabolites with longer gestations may also indicate better pregnancy health (e.g., reduced preterm birth). However, higher pre-implantation DEHP was also associated with a slower initial rise in hCG [33], a pattern that was also observed for women who had been exposed to diethylstilbestrol in utero [34], an exposure known to be associated with adverse reproductive outcomes [41]. This may indicate differences in the effect of DEHP exposure on different biological processes during early pregnancy. In this cohort, pre-implantation MCPP was associated with late corpus luteum rescue [33], indicative of poorer pregnancy health. MCPP was also associated with a shorter time from ovulation to implantation [33] one of the early pregnancy events that were associated with shorter gestations [10]. Consistent with these findings post-implantation MCPP was associated with earlier birth in our current analysis.

Studies that have examined the association between urinary phthalate metabolites and BPA during pregnancy and preterm birth have reported mixed results [18, 20, 21, 42], possibly due to differences in the exposure assessment. In the Study for Future Families (SFF), based on phthalate metabolite concentrations from a single spot urine, there was a reduced odds of preterm birth with increasing 3rd trimester DEHP metabolites [18]. In a nested case control study using the average of three phthalate metabolite measures across pregnancy (10, 18, and 26 weeks gestation) higher ∑DEHP metabolite concentrations were found to be associated with an increased risk of preterm birth [42]. Urinary concentrations from a single measure of BPA during the 3rd trimester were associated with increased risk of preterm birth in one study [20], but in another cohort that used the average BPA measured in 3 samples across pregnancy BPA was not associated with preterm birth [21]. Some studies examined length of gestation in relation to phthalate exposure [16, 18, 19, 43]. In a study of 3rd trimester phthalate metabolite concentrations and length of gestation, authors found shorter gestations were associated with higher concentrations of DEHP metabolites [19]. However, the SFF [18] found an association between 3rd trimester urinary DEHP metabolites and longer gestations, as did the Children’s Environmental Health Study [16], although not statistically significant. The latter two studies are consistent with our results but relied on a different timing of exposure assessment from ours. A study using preimplantation assessment showed associations between higher phthalate biomarker concentrations and shorter gestational length. The exception was for MEHP, where higher concentrations were associated with longer gestational length [43], consistent with our DEHP metabolites results.

Our study had limitations. The women who participated in the EPS were volunteers with no known fertility problems, the majority of whom were well-educated, white, non-smokers, and all of whom were planning a pregnancy, which may limit the generalizability of our results to more diverse populations. The urinary concentrations of phthalate metabolites measured in the EPS women were higher overall compared with concentrations measured in the U.S. general population during the 1999–2000 NHANES [3]. These differences may be a result of increased consumer awareness of the potentially endocrine disrupting properties of phthalates [44] resulting in manufacturing and behavior changes to reduce exposure compared to the 1980s when the EPS was conducted and may limit current applicability. The sample was small, which prevented us from assessing clinical preterm (*n* = 6) or post-term (*n* = 4) birth. However, we were able to assess time to birth among conceptuses that implanted, using implantation-based length of gestation. This time period is less variable than using an LMP-based gestational age [10], which is subject to variations in LMP to implantation among women. Finally, we carried out a number of tests reporting the association between biomarkers of 11 phthalate metabolites, a composite measure of the DEHP metabolites, and BPA with length of gestation. Given the exploratory nature of the study, we did not adjust for multiple comparisons and some of the observed associations may have occurred by chance.

Our study had several strengths. First, we have a very precise measure of gestational length, and we also account for data on medical interventions (induced labor and Caesarean delivery without labor) that artificially ended pregnancy early. We incorporated this information into our analysis allowing us to focus on natural length of pregnancy. We were also able to identify the day of implantation based on a highly sensitive assay for hCG [23], allowing us to examine length of gestation from implantation to birth, a characteristic unique to our study. Second, our exposure assessment data were based on biomarker concentrations in pooled samples. Concentrations of phthalate metabolites and BPA in pooled urine samples provide a more stable estimate of an individual’s typical exposure to these compounds which have short elimination half-lives. To reduce the potential for external contamination we measured phthalate metabolites and not the parent compounds, and for BPA we ruled out systematic contamination or degradation of the urine specimen by confirming that most BPA was present as a conjugate [26]. We also conducted pilot work that supported the stability of phthalate metabolites and BPA after long-term storage [24, 25]. We were able to use these pooled samples to characterize the pre-implantation and post-implantation early pregnancy biomarker measurements separately. Although our sample was small, we had little missing data, which allowed for almost complete exposure data.

## Conclusions

To our knowledge, this is the first study to examine pre-implantation and post-implantation early pregnancy urinary phthalate metabolite and BPA concentrations in relation to length of gestation in a cohort of naturally conceiving women. The associations of MEHHP with longer gestations and MCPP with shorter gestations raise the possibility that exposure to certain phthalates around the time of conception and during very early pregnancy could affect length of gestation. However, confirmation of these findings is needed, particularly given the small sample and multiple exposure biomarkers examined. Reproductive aged women continue to be exposed to compounds with potential endocrine disrupting properties, and those ongoing exposures warrant further investigation of their effects on pregnancy.

## Additional file

Additional file 1: **Table S1.** Spearman correlations between pre-implantation and post-implantation phthalate metabolites and bisphenol A. **Table S2.** Associations between pre-implantation and post-implantation early pregnancy biomarker concentrations and implantation-based length of gestation. **Figure S1.** Diagram of the urine samples pooled to measure phthalate metabolites and bisphenol A around the time of conception (pre-implantation measure). The sample period started the day after the end of menses and ended the day before implantation. Each sample was collected approximately 1 week apart and each woman (*n* = 125) contributed 3 samples to her pooled sample. **Figure S2.** Diagram of the urine samples pooled to measure phthalate metabolites and bisphenol A after embryo implantation (post-implantation measure). The sample period started the day after implantation and ended approximately 3 weeks later. Each sample was collected approximately 1 week apart and each woman (*n* = 121) contributed 3 samples to her pooled sample. **Figure S3.** Flowchart of participants from the Early Pregnancy Study who were included in the analysis of time from implantation to birth. (DOCX 75 kb)

## Data Availability

Data used in this study were from the North Carolina Early Pregnancy Study and Follow-up Study, Epidemiology Branch, NIEHS. The dataset used in this analysis is not publicly available due to the sensitive nature of the human subjects data.

## Abbreviations

BPA: Bisphenol A
CDC: Centers for Disease Control and Prevention
DEHP: di (2-ethylhexyl) phthalate
DES: diethylstilbestrol
EPS: Early Pregnancy Study
hCG: Human chorionic gonadotropin
LMP: Last menstrual period
MBP: mono-n-butyl phthalate
MBzP: monobenzyl phthalate
MCNP: monocarboxynonyl phthalate
MCOP: monocarboxyoctyl phthalate
MCPP: mono(3-carboxypropyl) phthalate
MECPP: mono(2-ethyl-5-carboxypentyl) phthalate
MEHHP: mono(2-ethyl-5-hydroxyhexyl) phthalate
MEHP: mono(2-ethylhexyl) phthalate
MEOHP: mono(2-ethyl-5-oxohexyl) phthalate
MEP: monoethyl phthalate
MiBP: mono-isobutyl phthalate
