# Supplementary material for: Association of urinary concentrations of early pregnancy phthalate metabolites and bisphenol A with length of gestation
Source: Environ Health. 2019 Aug 30;18:80. doi: 10.1186/s12940-019-0522-2 (PMC6717338; doi:10.1186/s12940-019-0522-2)
Supplement: Supplementary file 1 — Table S1. Spearman correlations between pre-implantation and post-implantation phthalate metabolites and bisphenol A. Table S2. Associations between pre-implantation and post-implantation early pregnancy biomarker concentrations and implantation-based length of gestation. Figure S1. Diagram of the urine samples pooled to measure phthalate metabolites and bisphenol A around the time of conception (pre-implantation measure). The sample period started the day after the end of menses and ended the day before implantation. Each sample was collected approximately 1 week apart and each woman (n = 125) contributed 3 samples to her pooled sample. Figure S2. Diagram of the urine samples pooled to measure phthalate metabolites and bisphenol A after embryo implantation (post-implantation measure). The sample period started the day after implantation and ended approximately 3 weeks later. Each sample was collected approximately 1 week apart and each woman (n = 121) contributed 3 samples to her pooled sample. Figure S3. Flowchart of participants from the Early Pregnancy Study who were included in the analysis of time from implantation to birth. (DOCX 75 kb) [file 12940_2019_522_MOESM1_ESM.docx]

Table S1. Spearman correlations between pre-implantation and post-implantation phthalate metabolites and bisphenol A

| **Exposure biomarker** | **Correlation between pre-implantation and post-implantation measures** |
| --- | --- |
| MBP | 0.36 |
| MEP | 0.47 |
| MBzP | 0.44 |
| MEHP | 0.59 |
| MEOHP | 0.35 |
| MEHHP | 0.29 |
| MECPP | 0.35 |
| MCNP | 0.22 |
| MCOP | 0.26 |
| MCPP | 0.60 |
| MiBP | 0.50 |
| ∑DEHP^a^ | 0.32 |
| BPA | 0.32 |
| ^a^∑DEHP represents the molar sum of 4 metabolites of DEHP: MEHP, MEHHP, MEOHP, and MECPP creatinine adjusted (nmol/mg creatinine). | |

Table S2. Associations between pre-implantation and post-implantation early pregnancy biomarker concentrations and implantation-based length of gestation.

|  | Pre-implantation | | | | Post-implantation | | | |
| --- | --- | --- | --- | --- | --- | --- | --- | --- |
| Biomarker^a^ | HR^b^ | 95%CI | | pvalue | HR | 95%CI | | pvalue |
| MBP | 0.81 | (0.53, | 1.26) | 0.35 | 0.91 | (0.59, | 1.41) | 0.68 |
| MEP | 1.01 | (0.66, | 1.56) | 0.95 | 0.93 | (0.60, | 1.44) | 0.75 |
| MBzP | 0.92 | (0.59, | 1.42) | 0.70 | 1.13 | (0.73, | 1.75) | 0.58 |
| MEHP | 0.88 | (0.57, | 1.35) | 0.55 | 0.96 | (0.62, | 1.48) | 0.84 |
| MEOHP | 0.74 | (0.47, | 1.15) | 0.18 | 0.78 | (0.50, | 1.21) | 0.26 |
| MEHHP | 0.55 | (0.35, | 0.86) | 0.01 | 0.87 | (0.56, | 1.35) | 0.53 |
| MECPP | 0.68 | (0.44, | 1.06) | 0.09 | 0.91 | (0.59, | 1.42) | 0.68 |
| MCNP | 0.82 | (0.53, | 1.27) | 0.37 | 0.97 | (0.62, | 1.50) | 0.89 |
| MCOP | 0.91 | (0.59, | 1.40) | 0.67 | 1.16 | (0.74, | 1.81) | 0.52 |
| MCPP | 1.20 | (0.78, | 1.84) | 0.41 | 1.59 | (1.02, | 2.49) | 0.04 |
| MiBP | 0.73 | (0.47, | 1.13) | 0.15 | 0.88 | (0.56, | 1.38) | 0.58 |
| ∑DEHP^c^ | 0.67 | (0.43, | 1.05) | 0.08 | 0.79 | (0.51, | 1.24) | 0.31 |
| BPA | 1.02 | (0.66, | 1.58) | 0.92 | 1.18 | (0.75, | 1.85) | 0.48 |
| ^a^Biomarker creatinine adjusted (ng/mg creatinine) concentrations from the pre-implantation (the time window between menses end and the day before implantation) and post-implantation (the time window starting the day after implantation).  ^b^HR: hazard ratios for risk of birth for women with biomarker concentrations above the median compared with those below the median.  ^c^∑DEHP represents the molar sum of 4 metabolites of DEHP: MEHP, MEHHP, MEOHP, and MECPP creatinine adjusted (nmol/mg creatinine). | | | | | | | | |

Figure S1. Diagram of the urine samples pooled to measure phthalate metabolites and bisphenol A around the time of conception (pre-implantation measure). The sample period started the day after the end of menses and ended the day before implantation. Each sample was collected approximately 1 week apart and each woman (n=125) contributed 3 samples to her pooled sample.


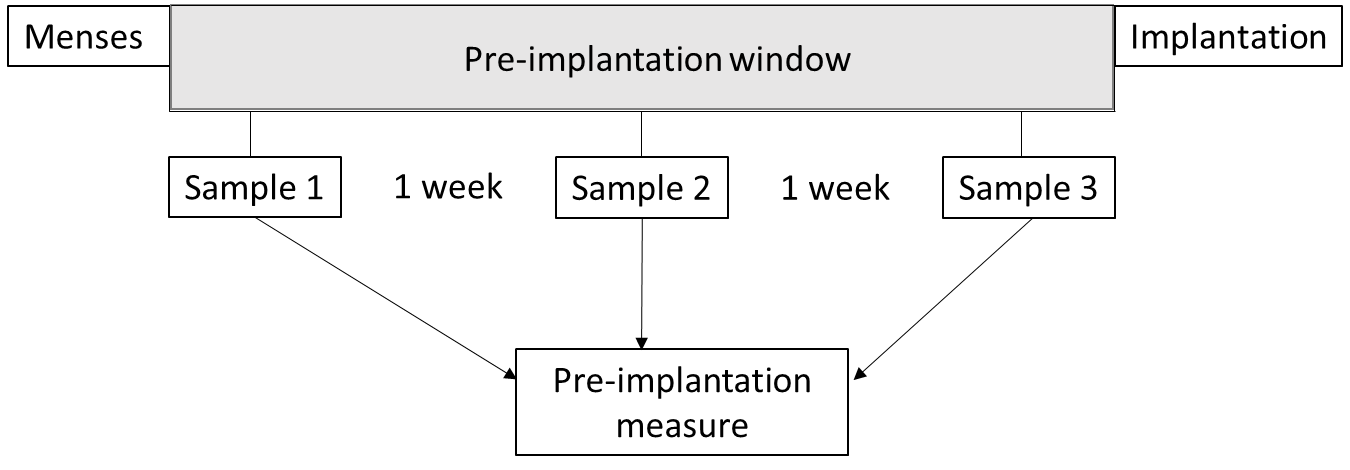


Figure S2. Diagram of the urine samples pooled to measure phthalate metabolites and bisphenol A after embryo implantation (post-implantation measure). The sample period started the day after implantation and ended approximately 3 weeks later. Each sample was collected approximately 1 week apart and each woman (n=121) contributed 3 samples to her pooled sample.


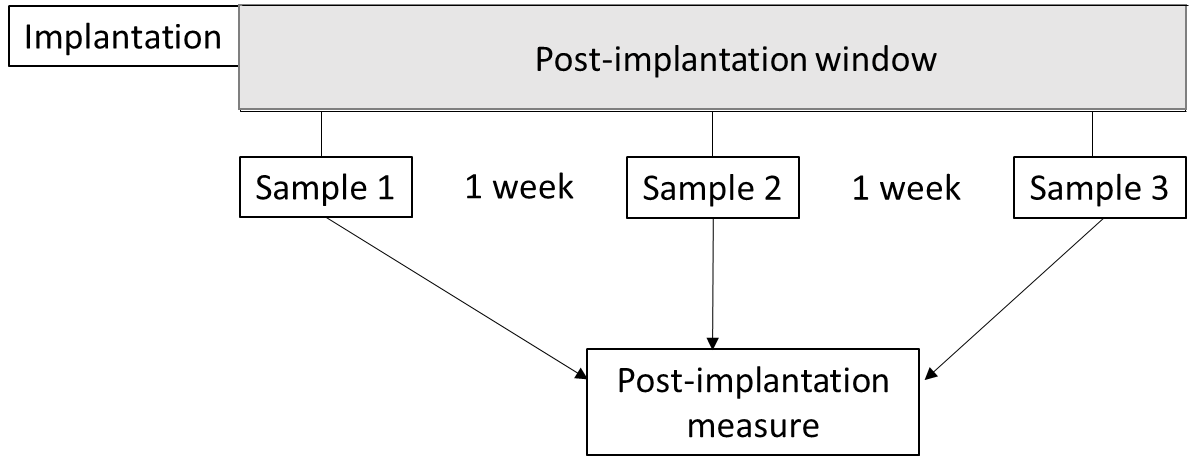


Figure S3. Flowchart of participants from the Early Pregnancy Study who were included in the analysis of time from implantation to birth.

6 sets of twins

151 clinical pregnancies

15 clinical losses

130 singleton live births

4 clinical pregnancies excluded because of DES exposure

**n=125 singleton live births in analysis**

1 clinical pregnancy missing phthalate and BPA measurement
